# The use of complementary and integrative health approaches for chronic musculoskeletal pain in younger US Veterans: An economic evaluation

**DOI:** 10.1371/journal.pone.0217831

**Published:** 2019-06-05

**Authors:** Patricia M. Herman, Anita H. Yuan, Matthew S. Cefalu, Karen Chu, Qing Zeng, Nell Marshall, Karl A. Lorenz, Stephanie L. Taylor

**Affiliations:** 1 RAND Corporation, Santa Monica, California, United States of America; 2 Center for the Study of Healthcare Innovation, Implementation and Policy, VA Greater Los Angeles Healthcare System, Los Angeles, California, United States of America; 3 Center for Health and Aging, VA Washington DC Healthcare System, Washington, District of Columbia, United States of America; 4 Biomedical Informatics Center, George Washington University, Washington, District of Columbia, United States of America; 5 Center for the Study of Innovation to Implementation, VA Palo Alto Healthcare System, Menlo Park, California, United States of America; 6 Stanford Medical School, Palo Alto, California, United States of America; 7 Department of Health Policy and Management, UCLA School of Public Health, Los Angeles, California, United States of America; University of Nebraska Medical Center, UNITED STATES

## Abstract

**Objectives:**

To estimate the cost-effectiveness to the US Veterans Health Administration (VA) of the use of complementary and integrative health (CIH) approaches by younger Veterans with chronic musculoskeletal disorder (MSD) pain.

**Perspective:**

VA healthcare system.

**Methods:**

We used a propensity score-adjusted hierarchical linear modeling (HLM), and 2010–2013 VA administrative data to estimate differences in VA healthcare costs, pain intensity (0–10 numerical rating scale), and opioid use between CIH users and nonusers. We identified CIH use in Veterans’ medical records through Current Procedural Terminology, VA workload tracking, and provider-type codes.

**Results:**

We identified 30,634 younger Veterans with chronic MSD pain as using CIH and 195,424 with no CIH use. CIH users differed from nonusers across all baseline covariates except the Charlson comorbidity index. They also differed on annual pre-CIH-start healthcare costs ($10,729 versus $5,818), pain (4.33 versus 3.76), and opioid use (66.6% versus 54.0%). The HLM results indicated lower annual healthcare costs (-$637; 95% CI: -$1,023, -$247), lower pain (-0.34; -0.40, -0.27), and slightly higher (less than a percentage point) opioid use (0.8; 0.6, 0.9) for CIH users in the year after CIH start. Sensitivity analyses indicated similar results for three most-used CIH approaches (acupuncture, chiropractic care, and massage), but higher costs for those with eight or more CIH visits.

**Conclusions:**

On average CIH use appears associated with lower healthcare costs and pain and slightly higher opioid use in this population of younger Veterans with chronic musculoskeletal pain. Given the VA’s growing interest in the use of CIH, further, more detailed analyses of its impacts are warranted.

## Introduction

Chronic pain is prevalent among Veterans in general,[1, 2] but especially among younger Veterans, including participants in Operation Enduring Freedom, Operation Iraqi Freedom and Operation New Dawn (OEF/OIF/OND).[3, 4] Musculoskeletal pain (e.g., back pain, neck pain, osteoarthritis) is the most common type of chronic pain.[5] This pain also often co-occurs with conditions such as anxiety, depression, post-traumatic stress disorder, and substance abuse.[3, 5]

Complementary and integrative health (CIH) approaches (therapies) such as yoga, meditation and acupuncture are being used for pain management in the Veterans Affairs (VA) healthcare system.[6, 7] CIH approaches are commonly provided to treat pain and its comorbidities, with acupuncture and chiropractic the most prevalent CIH approaches for pain.[8–10] A recent national survey found Veterans have substantial interest in and use of CIH approaches, with half reporting a use of a CIH approach in the prior year and 84% reporting interest in using CIH.[11]

Several guidelines recommend CIH approaches as effective for chronic pain,[12–15] and a few recent studies have shown these approaches to be associated with lower healthcare costs in the general population.[16–21] However, little is known about their impact on healthcare costs and utilization including opioid use in the VA where CIH is being increasingly emphasized. The VA has adopted the goal of implementing a Whole Health approach, in which CIH approaches are a key component.[22, 23]

We used VA administrative data to identify younger Veterans with chronic painful musculoskeletal disorders (MSD) who did and did not use CIH. We then estimated the impact of CIH use on VA healthcare costs, pain and opioid use to generate a preliminary estimate of the cost-effectiveness of CIH to the VA.

## Methods

### Chronic MSD pain cohort

The cohort for this study was drawn from a large previously-constructed cohort [24] of Veterans who used the VA’s healthcare system during the 2010–2013 period with at least one visit with a MSD International Classification of Disease, Version 9, Clinical Modification (ICD-9-CM) diagnosis code. To identify MSD, we used 1,685 ICD-9-CM MSD codes assembled from previous reviews and team input, as well as text downloaded from members’ medical records.

From this larger cohort, we identified younger Veterans who would have been 18 to 40 years of age at the time of the OEF/OIF/OND conflicts (i.e., those born 1960–1995). We then identified those with chronic MSD pain using either: 1) 2 or more occurrences ≥30 days apart of MSD ICD-9-CM codes from the “likely to represent chronic pain” list from a study on identifying chronic pain from electronic records (S1 Appendix);[25] or 2) 2 or more occurrences of common MSD ICD-9-CM codes plus 2 or more self-reported pain scores ≥4 on a 0 (no pain) to 10 (maximum pain) scale, both within 90 days.

As we aimed to examine the impact of CIH use on VA healthcare users, and on healthcare utilization outcomes that could be influenced by short-term CIH use (versus utilization due to a high-cost catastrophic event), we eliminated those with $0 or >$50,000 annual costs,[26] and those who started CIH during a hospital stay.

### CIH user group, control group, and start dates

From this younger-Veteran chronic MSD pain cohort we defined CIH users as users of one or more of nine CIH approaches which could be identified from codes available in data from the VA’s Corporate Data Warehouse (CDW).[27] The codes used to identify CIH use were: Current Procedural Terminology (CPT) codes for the five types of CIH with these codes (i.e., acupuncture, biofeedback, chiropractic care, hypnosis/hypnotherapy, and massage); CHAR4 codes (used to track workload in the VA system) for the eight types of CIH that have these codes (i.e., acupuncture, biofeedback, guided imagery, hypnosis/hypnotherapy, therapeutic massage, meditation, Tai Chi, and yoga); and provider-type codes for chiropractors. We only considered CIH use that started after entry into the chronic MSD pain cohort, and to ensure we identified initial CIH use, we dropped those who used CIH during the first six months of 2010.

The control group included all Veterans in the cohort with no CIH codes, and no indications of CIH-related key words in their medical records. We defined the start dates for the CIH group as the first CIH use documented in each Veteran’s medical record as part of an outpatient visit using CPT, CHAR4 or chiropractic provider-type codes. Controls lack comparable “start dates” for CIH initiation, but to assure we compared their outcomes during a similar period in their healthcare history, we first randomly assigned the control group pseudo-start dates following the same general distribution as seen in the CIH user group in terms of days between qualifying for the MSD cohort and CIH start. We then identified the nearest (+/-90 days) outpatient visit and set that as the control group member’s “start date” to ensure both groups had start dates that were roughly the same length of time after entry into the MSD cohort and associated with an outpatient visit. If a member of the control group did not have an outpatient visit near their randomly assigned pseudo-start date, we dropped them from the cohort. The control group’s start dates were only used to define the time periods for their year-before and year-after outcomes.

### Outcomes

For each outcome, we captured data one year before and one year after each cohort-members’ start date.

#### Healthcare costs

We derived healthcare utilization data from the VA’s Medical SAS Inpatient and Outpatient Datasets, which contain VA inpatient, outpatient and pharmacy services use, including the use of CIH approaches. We obtained estimates of the cost of each type of care from the average cost database of the VA’s Health Economics Resource Center (HERC).[28–30] The average cost database allows VA healthcare utilization to be valued at costs that are similar to Medicare payment rates. Payments for inpatient, outpatient and pharmacy care provided by non-VA providers, but authorized and paid for by VA, were captured from the VA Fee Basis files.[31] Costs incurred in each year were converted 2013 costs using the appropriate Consumer Price Index (CPI).[32]

#### Pain intensity

A 2000 VA initiative established pain as the “fifth vital sign” and began requiring regular documentation of Veterans’ reported pain on a 0–10 numerical rating scale (NRS) in clinical settings where vital signs are recorded.[33] This policy and its adherence allow these pain scores to provide a reasonable estimate of the health effects of interventions targeting painful conditions. We captured reported pain intensity for our chronic MSD cohort from the VA CDW Vital Signs Tables. This measure of pain is simple and has been extensively studied.[34–38] When multiple pain scores were recorded during a day, we used the highest score.[24]

#### Opioid use

We used VA pharmacy data to categorize opioid use according to the scheme outlined by a panel of experts in opioid therapy and VA pain management practice and policy.[39] We defined opioid users as those for whom at least one outpatient opioid prescription for an oral or transdermal formulation was dispensed from a VA pharmacy in the period.

#### Covariates

We utilized 32 covariates extracted from the VA CDW and Vital Status files in our analyses. For demographics we captured age in years at start date (18–34, 35–44 and 45–54 years), gender, race/ethnicity (non-Hispanic white, non-Hispanic black, Hispanic, non-Hispanic other), and marital status (married, single/never married, divorced/separated/widowed). We captured the following data on Veterans’ interaction with the healthcare system: financial means test (copay required or copay exempt based on an annual financial assessment), service connectedness (the extent to which any disability was caused by, incurred during, or aggravated by military service; <50 percent, ≥50 percent), and eligibility priority group (priority group 1, priority group 2–6, priority group 7–8, see website for details[40]). We captured the type of MSD pain in our chronic MSD pain cohort using ICD-9-CM codes for back pain, neck pain, joint pain, osteoarthritis, temporomandibular disorder, and fibromyalgia. We also used ICD-9-CM codes to capture data on 6 conditions commonly associated with chronic pain: anxiety, depression, post-traumatic stress disorder (PTSD), traumatic brain injury (TBI), sleep disturbance, and substance abuse. Finally, we captured Veterans general health on a number of dimensions commonly used in propensity scores using the categories and codes defined by the Agency for Healthcare Research and Quality’s Clinical Classification Software (CCS).[41] The CCS codes are groups of ICD-9-CM codes and are defined for asthma, cancer, cerebrovascular disease, chronic obstructive pulmonary disease, coronary artery disease, diabetes, headache, HIV infection, hypertension, mental illness, and obesity. We also calculated the Charlson comorbidity index.[42]

### Analyses

Our outcome data are available for multiple points in time over two years for each Veteran, and these outcomes occurred within the VA facility where the Veteran received care. Thus, the outcome data are likely correlated both within Veteran and within VA facility, and as such, do not meet the usual independence assumptions needed for traditional regression models.[26] Hierarchical linear modeling [43–45] (HLM) corrects for the error structure violations that can be caused by methods that require each observation’s errors to be independent.[43, 46] HLM also allows for unbalanced designs (e.g., different numbers of observations in the subgroups studied) which are troublesome to other methods,[43, 47] and because it uses all information available in the dataset, optimizes estimation in the presence of randomly missing data.[44, 47] Finally, the specification of the HLM allows for a different intercept and linear time effect for each of the following three groups: CIH users before CIH start, CIH user after CIH start, and a control group. The flexibility of this model specification allowed us to examine differences between the control groups and CIH users before the start of CIH and the impact of CIH use on CIH users after CIH start. Below we discuss how we used propensity score weighting to create a control group, and a good propensity score weight in this context would be indicated by near zero differences in the intercept and time (slope) coefficients between the control group and CIH users before CIH start. However, even in the face of an insufficient control group, the HLM allows for estimation of the impact of CIH use on CIH users by comparing the intercept and time coefficients of CIH users before and after CIH start. That is, the HLM leverages the within-individual change in outcomes over time to assess the impact of CIH use.

Because Veterans’ use of CIH was self-selected systematic differences in outcomes may exist between those who do and do not use CIH that are not due to the CIH use itself.[48–57] Instead of directly adjusting for these differences in the HLM by including covariates, we opted to use a propensity score adjustment. Propensity scores were originally introduced as a way to generate an unbiased estimate of the (causal) effect of an exposure (e.g., CIH use) in the presence of confounding.[58] In brief and ideally, propensity scores have the ability to replicate the benefits of a randomized trial by allowing comparison across those with the same probability of exposure (same propensity score) but who did and did not experience the exposure.[59] Propensity scores were estimated using logistic regression predicting CIH use from the covariates and baseline values of each outcome (healthcare costs, pain, and opioid use) in the year before the CIH start date. Because the availability of CIH varied by facility we did not include facility in the propensity scores. The propensity scores were subsequently used to weight the HLM so that those who do not use CIH (the control group) are as similar as possible (except for the CIH use itself) to those that used CIH. The propensity score weights were derived as ‘1’ for CIH users and the inverse of the propensity score for the controls. This allowed us to estimate the average treatment effect on the treated (ATT).

#### Data preparation

For the HLM analysis we used monthly costs, daily pain scores, and monthly opioid use for the 12 months before and after each member’s start date. We used enrollment files to identify Veterans who had less than one year of data before their start date and dropped those with less than 180 days. We also dropped anyone with no pain scores within a year. Opioid use was measured as the release to the Veteran of any opioid prescription during a month. Months without costs and without opioid use were coded as zero costs and zero opioid use, respectively, while days without pain measurements were treated as missing data. All monthly or daily data points before a Veteran’s qualifying date (first MSD diagnosis) were dropped from the analysis.

#### Sensitivity analyses

The CIH user group in our primary analyses included all cohort members with at least one visit for a CIH-coded approach. The three most common types of CIH used were acupuncture, chiropractic care and massage, and some consider a therapeutic dose to be 8 or more treatments.[60–63] Our sensitivity analyses examined healthcare cost, pain and opioid use for four subgroups: those who used any number of visits for acupuncture, massage, and chiropractic care, and those who had eight or more CIH visits of any kind.

#### Cost-effectiveness analysis

Our cost-effectiveness analyses used the perspective of the VA. For the main analysis, we compared the difference in total annual VA healthcare costs to the difference in pain over the year after CIH start for those using any type and amount of CIH. Opioid use was considered a secondary outcome. Our one-year outcomes made discounting unnecessary. All analyses were run using STATA 15, StataCorp, College Station, Texas.

This study was approved by the Department of Veterans Affairs, Greater Los Angeles Healthcare System, Institutional Review Board, and by the VA GLA Associate Chief of Staff, Research and Development, R&D Committee, and all required subcommittees. This manuscript conforms to the Consolidated Health Economic Evaluation Reporting Standards (CHEERS). See S1 Table.

## Results

**Fig 1:**
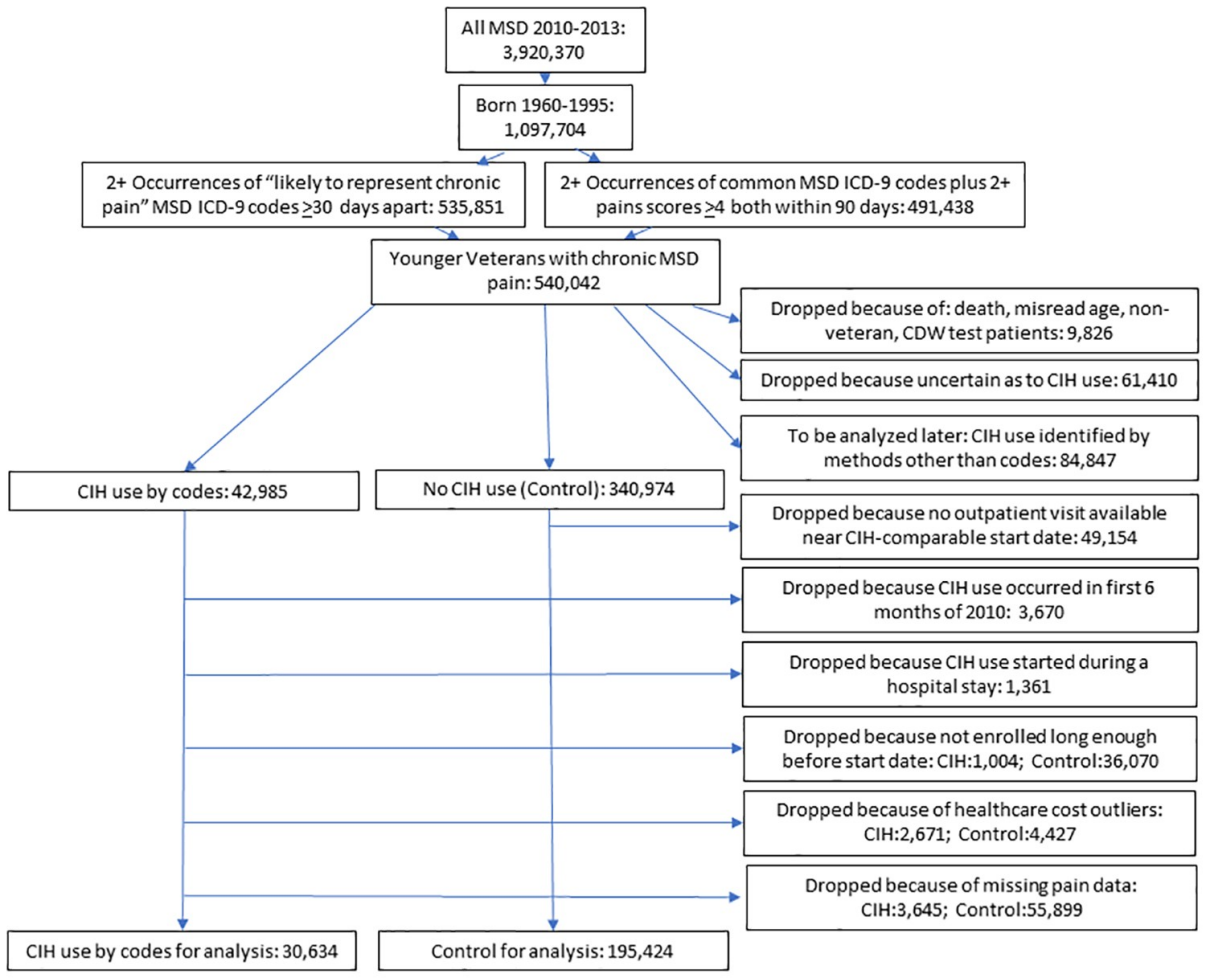
Veteran flow into the analytic cohort. CDW = Corporate Data Warehouse (repository for US Veterans Health Administration data); CIH = complementary and integrative health approaches/therapies; MSD = musculoskeletal disorder.

The steps involved in building our analytic cohort are shown in Fig 1. As can be seen, applying either of our two chronic MSD diagnosis criteria alone produced similar results: 99.2% (535,851/540,042) of our cohort met the first criterion and 91.0% (491,438/540,042) met the second. Further cleaning resulted in a final analytic cohort of 30,634 CIH users and 195,424 nonusers (control).

Table 1 shows the values for each covariate and for healthcare costs, pain intensity and opioid use in the year before start date for each group. As can be seen, the groups differed significantly on all patient characteristics other than the Charlson comorbidity index. The last three columns provide balance diagnostics on the propensity score weighting. The propensity score weighting improves the similarity of the groups. The average absolute standardized difference decreased after weighting to less than 0.01 in absolute value for all but joint pain and year-before healthcare costs and opioid use, and ratios of the variances between groups after weighting were within 0.02 of 1.0 for all but fibromyalgia, and year-before pain and healthcare costs.

**Table 1:** Covariates available for analysis, their values in each group, and the impact of propensity score weighting on the differences seen in means and variances.

	CIH code group (n = 30,634)	Control group before weighting (n = 195,424)	p-value	Standardized Difference		Var† ratio
				Raw	Wtd*	Wtd*
Demographics						
Female (Ref: Male)	7,360 (24.0%)	31,934 (16.3%)	< .001	0.192	0.003	1.004
Age (Ref: 18–34 years)						
Age 35–44 years	10,148 (33.1%)	62,260 (31.9%)	< .001	0.027	0.001	1.001
Age 45–54 years	10,738 (35.1%)	80,105 (41.0%)	< .001	-0.123	-0.002	0.999
Race/Ethnicity (Ref: Non-Hispanic White)						
Non-Hispanic Black	6,377 (20.8%)	51,011 (26.1%)	< .001	-0.125	0.010	1.015
Hispanic or Latino	4,364 (14.3%)	15,471 (7.9%)	< .001	0.203	0.001	1.002
Non-Hispanic Other	1,332 (4.4%)	6,613 (3.4%)	< .001	0.050	-0.002	0.992
Marital status (Ref: Married)						
Single/Never married	7,500 (24.5%)	42,815 (21.9%)	< .001	0.061	0.003	1.004
Divorced/Separated/Widowed	9,008 (29.4%)	55,939 (28.6%)	0.005	0.017	-0.004	0.997
Missing marital	66 (0.2%)	524 (0.3%)	0.093	-0.011	0.001	1.013
Veteran status and interaction with the VA healthcare system						
Financial means test (Ref: Copay exempt)						
Copay required	2,183 (7.1%)	21,157 (10.8%)	< .001	-0.130	-0.001	0.995
Other financial means test	877 (2.9%)	8,434 (4.3%)	< .001	-0.078	0.002	1.011
Health insurance status (Ref: VA coverage only)						
Private insurance	6,080 (19.9%)	45,586 (23.3%)	< .001	-0.085	0.006	1.009
Non-VA government insurance	5,831 (19.0%)	28,685 (14.7%)	< .001	0.117	-0.005	0.992
Eligibility for VA benefits (Ref: Priority group 1)						
Priority group 2–6‡	15,409 (50.3%)	114,162 (58.4%)	< .001	-0.164	0.005	1.000
Priority group 7–8‡	1,093 (3.6%)	13,181 (6.7%)	< .001	-0.144	-0.003	0.985
Missing priority group	85 (0.3%)	458 (0.2%)	0.152	0.009	-0.001	0.988
Whether disability connected to military service (Ref: Service connectedness <50%)						
Service connectedness ≥50%§	16,250 (53.1%)	77,557 (39.7%)	< .001	0.270	-0.002	1.000
Missing service connectedness§	3,418 (11.2%)	38,135 (19.5%)	< .001	-0.233	-0.005	0.988
Pain-related comorbidities						
Anxiety	8,646 (28.2%)	37,831 (19.4%)	< .001	0.209	-0.001	0.999
Depression	16,724 (54.6%)	74,534 (38.1%)	< .001	0.335	-0.001	1.000
Post-traumatic stress disorder	13,862 (45.3%)	58,147 (29.8%)	< .001	0.324	0.004	1.001
Traumatic brain injury	4,416 (14.4%)	13,767 (7.0%)	< .001	0.240	-0.001	0.997
Substance abuse	13,384 (43.7%)	80,137 (41.0%)	< .001	0.054	-0.006	0.998
Sleep disturbance	9,196 (30.0%)	47,343 (24.2%)	< .001	0.131	-0.001	0.999
Types of musculoskeletal disorders						
Back pain	17,859 (58.3%)	97,441 (49.9%)	< .001	0.170	-0.007	1.002
Neck pain	6,855 (22.4%)	29,710 (15.2%)	< .001	0.184	-0.009	0.988
Joint pain	9,243 (30.2%)	79,249 (40.6%)	< .001	-0.218	0.012	1.011
Osteoarthritis	2,180 (7.1%)	16,661 (8.5%)	< .001	-0.053	-0.002	0.994
Temporomandibular joint disorder	236 (0.8%)	863 (0.4%)	< .001	0.042	0.001	1.010
Fibromyalgia	2,461 (8.0%)	13,227 (6.8%)	< .001	0.048	0.007	1.021
General comorbidities						
Human immunodeficiency virus	204 (0.7%)	1,301 (0.7%)	< .001	0.001	-0.001	0.990
Cancer	3,713 (12.1%)	18,367 (9.4%)	< .001	0.088	-0.003	0.992
Diabetes	4,909 (16.0%)	35,296 (18.1%)	< .001	-0.054	-0.001	0.999
Obesity	12,403 (40.5%)	73,397 (37.6%)	< .001	0.060	0.005	1.001
Headache	13,370 (43.6%)	60,205 (30.8%)	< .001	0.268	-0.002	0.999
Hypertension	10,724 (35.0%)	75,358 (38.6%)	< .001	-0.074	0.000	1.000
Heart disease	10,493 (34.3%)	59,083 (30.2%)	< .001	0.087	-0.006	0.996
Cerebrovascular	867 (2.8%)	4,553 (2.3%)	< .001	0.032	-0.000	0.999
Chronic obstructive pulmonary disease	4,393 (14.3%)	24,213 (12.4%)	< .001	0.057	-0.007	0.986
Asthma	3,522 (11.5%)	17,974 (9.2%)	< .001	0.076	-0.003	0.993
Mental illness	26,873 (87.7%)	157,388 (80.5%)	< .001	0.198	0.001	0.998
Charlson index‖	4,893 (16.0%)	30,394 (15.6%)	0.060	0.012	-0.005	0.991
Year-before the start date values for each outcome						
Annual healthcare cost	$10,729 ($9,562)	$5,818 ($6,589)	< .001	0.598	-0.070	0.639
Time-weighted pain score	4.33 (2.3)	3.76 (2.6)	< .001	0.232	-0.005	0.887
Opioid use	20,408 (66.6%)	105,516 (54.0%)	< .001	0.268	-0.012	1.009

*Wtd = weighted using propensity scores.

^†^Variance ratio = ratio of the variances seen in each group. Ideally, after weighting this number should be near 1.0.

^‡^Eligibility priority group based on a Veteran’s service, service-related disability, honors, and income: priority group 1 (highest), priority group 2–6, priority group 7–8.

^§^Service connected disability was incurred or aggravated during active military service. The percentage relates to the level of disability incurred.

^‖^Based on Quan H, Sundararajan V, Halfon P, et al. Coding algorithms for defining comorbidities in ICD-9-CM and ICD-10 administrative data. Med Care. 2005: 1130–39.

Table 2 reports the results of the HLM models. The negative significant coefficients for time indicated that healthcare costs, pain and opioid use all generally decrease over the two-year period for the control group. The significant CIH user coefficients indicated that the CIH group had significantly higher healthcare costs and pain and lower opioid use to start. The positive and significant CIH x time interaction coefficients indicated that pre-CIH start trajectories are different between groups, and the size of these coefficients is such that the CIH group experienced less of a reduction in healthcare costs and a net increase in pain and opioid use in the year before CIH start. Therefore, despite the propensity score weighting the two groups have different levels of patterns of healthcare costs, pain or opioid use in the year before CIH start. Among CIH users there was a significant downward shift in the trajectories for healthcare costs and pain and significant upward shift in opioid use at CIH start (main effect of CIH start) and minor changes in slope thereafter (interaction of CIH start and time). Average monthly healthcare costs by general cost category for the CIH group in the years before and after the start date are shown in S1 Fig. The significant random effects for facility indicated that outcomes are clustered by facility. Initial model runs also indicated a small random effect on facility slope. However, this term was excluded from later models because it added little to explanatory power and significantly hampered model convergence. As shown at the bottom of the table, and according to the estimated coefficients, CIH use was associated with a significant decrease in healthcare costs and pain, and a less than a percentage point increase in opioid use compared to control.

**Table 2:** Results of the hierarchical linear models (HLM) for each outcome over the 2-year study period.

	Healthcare costs ($000)		Pain (0–10 scale)		Opioid use (proportion using)	
	Estimate (SE)	p-value	Estimate (SE)	p-value	Estimate (SE)	p-value
Intercept	0.9960 (0.019)	< .001	4.2556 (0.031)	< .001	0.3254 (0.005)	< .001
Time	-0.0256 (0.001)	< .001	-0.0004 (0.000)	< .001	-0.0018 (0.000)	< .001
CIH user	0.0599 (0.031)	0.054	0.1795 (0.044)	< .001	-0.0394 (0.005)	< .001
CIH x time	0.0125 (0.001)	< .001	0.0008 (0.000)	< .001	0.0051 (0.000)	< .001
CIH start	-0.0510 (0.013)	< .001	-0.3527 (0.033)	< .001	0.0598 (0.004)	< .001
CIH start x time	-0.0003 (0.001)	0.584	0.0001 (0.000)	0.047	-0.0044 (0.000)	< .001
Random effects parameters						
Facility intercept	0.0239 (0.002)	< .001	0.2433 (0.034)	< .001	0.0058 (0.001)	< .001
Individual intercept	0.3352 (0.007)	< .001	2.7490 (0.061)	< .001	0.1399 (0.003)	< .001
Individual slope	0.0000 (0.000)	< .001	0.0000 (0.000)	< .001	0.0003 (0.000)	< .001
Residual	3.0325 (0.076)	< .001	6.9369 (0.100)	< .001	0.0732 (0.001)	< .001
Estimated annual change (95% CI)	-$637* (-$1,023, -$247)		-0.34 (-0.40, -0.27)		0.76† (0.59, 0.93)	

*In 2013 USD

^†^Percentage point change

Table 3 gives the HLM results for four CIH subgroups included in our sensitivity analyses. The models for acupuncture, chiropractic care and massage all show a reduction in healthcare costs and pain and a small increase in opioid use. The subgroup with 8+ visits had a similar reduction in pain and increase in opioid use, but with an increase in healthcare costs.

**Table 3:** Results of the sensitivity analyses.

	Healthcare costs (USD) Estimate (95% CI)	Pain (0–10 scale) Estimate (95% CI)	Opioid use (percentage points) Estimate (95% CI)
Base case—any use of CIH (n = 30,634)	-$637 (-$1,023, -$247)	-0.33 (-0.40, -0.27)	0.76 points (0.59, 0.93)
8 or more CIH visits (n = 5,053)	$1,465 ($693, $2,236)	-0.29 (-0.43, -0.19)	0.86 points (0.52, 1.19)
Acupuncture (n = 7,694)	-$216 (-$972, $539)	-0.20 (-0.31, -0.11)	0.63 points (0.34, 0.92)
Chiropractic (n = 15,836)	-$212 (-$604, $181)	-0.35 (-0.47, -0.28)	0.73 points (0.54, 0.92)
Massage (n = 7,604)	-$697 (-$1,486, $92)	-0.35 (-0.50, -0.22)	0.67 points (0.43, 0.91)

CIH = complementary and integrative health approaches/therapies.

## Discussion

This study used hierarchical linear modeling to examine the effect of the use of CIH approaches on VA healthcare costs, pain intensity and opioid use in a cohort of younger Veterans with chronic MSD who use VA healthcare services. The results showed lower healthcare costs and reduced pain for Veterans who used CIH: an average of $637 lower healthcare costs and 0.34 lower pain intensity on the 0–10 NRS. The estimates also revealed a 0.76 percentage point increase in the rate of opioid use for the year after onset of CIH use.

As expected, because each make up a substantial proportion of CIH use, the results seen for acupuncture, chiropractic care and therapeutic massage users were similar to those seen for users of any of the nine CIH approaches examined. Those who had 8+ visits for CIH during the year had similar reductions in pain and increases in opioid use, but an increase in healthcare costs of almost $1,500 per year. This increase in costs could partially be explained by the cost of the additional CIH visits. However, half of those with 8+ visits had 8–10 visits and all but 1 percent had fewer than 30 visits. The cost increase could also be related to how Veterans use VA healthcare. A recent analysis of Medical Expenditures Panel Survey (MEPS) data found that in 2014–2015 Veterans who used the VA healthcare system had higher overall costs than non-VA users and that only one-third of the total healthcare received by VA users was paid by the VA.[64] Our healthcare cost and CIH codes only captured utilization that occurred at or was reimbursed by the VA; we could be missing costs and CIH use that happened completely or partially outside the VA.

A number of systematic reviews have shown that CIH use is associated with reductions in MSD pain.[12, 13, 65–76] The reduction in pain intensity of 0.34 points on a 0–10 scale may seem clinically insignificant. However, these pain scores were captured not as outcomes in a clinical trial, but at admission during hospital stays and primary care visits when the Veteran could be experiencing other non-MSD pain or have made the visit because of a pain flare-up. The fact that these scores decreased significantly after CIH start is one indication of potential CIH benefit. Given that pain scores are only captured when Veterans interact with the health system, the reduction in healthcare utilization implied by the reduction in healthcare costs might also be considered evidence of reduced pain in CIH users. Little is known about the impact of CIH use on opioid use. One theory for the small increase in opioid use among CIH users is that their CIH use onset might have occurred after trying many other pain management options, with CIH use as a last resort. Further examination of the relationship between and timing of CIH and opioid use may be worthwhile.

Two other studies found cost savings across individuals using a variety of CIH approaches.[17, 21] The first used 2000–2003 data from two large insurers in Washington State (where coverage was mandated for all licensed providers) to examine the impact of CIH use for back pain.[17] CIH use was defined as at least one visit to a chiropractor, licensed massage therapist, acupuncturist, or naturopathic physician during the year after diagnosis. This study used frequency matching between CIH users and nonusers based on gender, 10-year age group, total expenditures prior year, and disease burden, and found an average reduction in healthcare costs of $329 per plan member with back pain in the year after diagnosis.

The second study used 2002–2008 MEPS data to examine the impact of CIH use on spinal pain.[21] CIH users were defined as having at least one visit to a chiropractor, massage therapist, homeopathic provider, acupuncturist, or “other CAM provider.” In propensity score-matched samples average expenditures for the CIH users were $526 lower for spine-specific costs and $298 lower for total health costs.

Other studies have examined healthcare costs for those who use acupuncture and chiropractic care. A study of the use of acupuncture in Alberta for low back pain age- and gender-matched those who used acupuncture to those who did not found that physician visits decreased by almost half in the year after acupuncture start, the cost of these visits dropped by 37%, and that the cost reductions were larger for those with 10+ acupuncture visits during the year.[19] Another study examining the effect of having a chiropractic coverage benefit on healthcare utilization and costs found that compared to members with healthcare use, but without chiropractic coverage, those with use and this coverage had an average of $208 lower overall healthcare costs per year during 2000.[20] Finally, a study of back-related healthcare costs for those using chiropractic or medical providers to manage their low back pain during a 2-year period 2004–2005 found that the average back-related cost of chiropractic treatment was $1,933 less over the 2-year period than medical care.[18]

This study benefits from a large real-world cohort and the use of administrative data. However, it also has several limitations, including all the limitations inherent in a cohort study attempting to create comparison groups that only differ by CIH use. The only variables available for adjusting between groups were those in administrative data, and although we included 32 covariates, they were insufficient to create an adequate balance between the groups at baseline. Therefore, it is likely that the differences seen between groups in this study were not all attributable to CIH use. We used a commonly used, well-accepted and understood approach (logistic regression) to estimate our propensity score weights. However, other approaches (e.g., covariate balancing propensity scores[77] or machine learning[78]) are available and may have produced different results. Nevertheless, because of our modeling approach we were able to estimate of the impact of CIH use on CIH users. To best identify those who used CIH for their chronic MSD we eliminated those who used CIH before they qualified as having chronic MSD. Thus, our estimates should not be assumed to apply to long-term CIH users. Our HLM models assumed linear trends when healthcare costs, pain and/or opioid use over time might not be linear. We did allow a one-time change at CIH start followed by a change in trajectory for those in the CIH group. However, other than this pattern of rapid change in the first weeks of treatment followed by more gradual change seen in many trials of CIH, we had no hypotheses as to actual trajectory shape, so left the models as linear. Our healthcare cost data only captured healthcare utilization that occurred at or was reimbursed by the VA, which according to a recent study of MEPS data only accounts for about one-third of the total healthcare received by VA users.[64] Therefore, we could be missing healthcare costs and CIH use that happened completely or partially outside the VA system. The pain scores were assumed to be due to Veterans’ chronic MSD. However, since these pain scores were captured in healthcare encounters and not as outcomes in a clinical trial they could be due to other painful conditions. They also likely understate pain reductions over time since high-pain events would be more likely than lower pain states to lead to healthcare encounters where pain would be recorded. We were limited to data from 2010–2013 because this previously assembled dataset contained a full set of medical records. It is also worth noting that our data span the height of opioid prescribing at the VA.[79] Analysis of more recent data may show different patterns in CIH, opioid, and other healthcare use.

## Conclusions

According to our propensity score-weighted HLM analysis, in a cohort of younger Veterans with chronic MSD pain during 2010 through 2013, any use of CIH was not only cost-effective, it was cost saving. Any use of CIH was associated with an average reduction in healthcare costs of $637, a 0.34-point reduction in pain intensity on a 0–10 pain scale, and a less than one percentage point increase in opioid use during the year after CIH start. Before CIH start, CIH users’ healthcare costs and pain are different than those of nonusers in ways that are not explained by data available in administrative records on demographics, socioeconomic factors, comorbidities, and MSD pain type. Care should be taken in future studies of CIH use using propensity scores in this population to ensure their adequacy in balancing groups. Also, given the prevalence of and increasing interest in CIH use in the VA, additional analyses should be undertaken to confirm these results on more recent data, and to understand the healthcare cost impacts of high-CIH utilizers.

## Supporting information

S1 Appendix Musculoskeletal ICD-9 codes likely to represent chronic pain and used as one criterion to identify Veterans with chronic musculoskeletal pain. Source: Tian TY, Zlateva I, Anderson DR. Using electronic health records data to identify patients with chronic pain in a primary care setting. Journal of the American Medical Informatics Association. 2013; 20: e275-e80.
(DOCX)

S1 Table CHEERS Checklist: Items to include when reporting economic evaluations of health interventions. (DOC)

S1 Fig Graphs of average monthly healthcare costs in the CIH group in the months before and after CIH start. These graphs will not exactly match the results from our model because monthly averages across Veterans, which is what is shown here, are not equivalent to average trends across individual Veterans, which is what our model estimates. However, these graphs are offered to help clarify the healthcare cost components involved. As can be seen, the bulk of healthcare costs are outpatient costs and although some reduction is seen in inpatient costs, the main changes pre- to post-CIH start seem to occur in outpatient costs. Nevertheless, our model was estimated using total costs.
(PPTX)
