# Supplementary material for: The use of complementary and integrative health approaches for chronic musculoskeletal pain in younger US Veterans: An economic evaluation
Source: PLoS One. 2019 Jun 5;14(6):e0217831. doi: 10.1371/journal.pone.0217831 (PMC6550429; doi:10.1371/journal.pone.0217831)
Supplement: S1 Appendix — Source: Tian TY, Zlateva I, Anderson DR. Using electronic health records data to identify patients with chronic pain in a primary care setting. Journal of the American Medical Informatics Association. 2013; 20: e275-e80. (DOCX) [file pone.0217831.s001.docx]

**Appendix. Musculoskeletal ICD-9 codes likely to represent chronic pain and used as one criterion to identify Veterans with chronic musculoskeletal pain**

719.41 Joint Pain, Shoulder

719.49 Joint Pain Multiple Sites

719.45 Joint Pain, Hip or Thigh

719.46 Pain Joint - Lower Leg, Knee

719.47 Joint Pain - Ankle

720 Anklosing Spondylitis

720.2 Inflammation of Sacroiliac Joint, Not otherwise specified

720.9 Inflammatory Spondylopathy, Not otherwise specified

721 Cervical Arthritis

721.1 Anterior Spinal Artery Compression Syndrome

721.2 Thoracic Arthritis

721.3 Lumbar and Sacral Arthritis

721.41 Spondylogenic Compression of Thoracic Spinal Cord

721.42 Lumbar Spondylosis with Myelopathy

721.6 Ankylosing Vertebral Hyperostosis

721.8 Other Allied Disorders of Spine

721.9 Degenerative Spinal Arthritis

721.91 Spondylogenic Compression of Spinal Cord, Not otherwise specified

722 Cervical Disc Displacement

722.1 Displacement of Lumbar Intervertebral Disc without Myelopathy

722.11 Displacement of Thoracic Intervertebral Disc without Myelopathy

722.2 Disc Displacement, Not otherwise specified

722.3 Schmorl's Disease

722.31 Schmorl's Nodes of Thoracic Region

722.32 Schmorl's Nodes of Lumbar Region

722.39 Schmorl's Nodes of Other Region, Not elsewhere classified

722.4 Cervical Disc Degeneration

722.51 Degeneration of Thoracic Intervertebral Disc

722.52 Degeneration of Lumbar Intervertebral Disc

722.6 Degeneration of Intervertebral Disc, Not otherwise specified

722.7 Disc Disease with Myelopathy, Not otherwise specified

722.71 Cervical Disc Disease with Myelopathy

722.73 Intervertebral Disc Disorder of Lumbar Region with Myelopathy

722.8 Postlaminectomy Syndrome, Not otherwise specified

722.81 Postlaminectomy Syndrome - Cervical

722.82 Postlaminectomy Syndrome - Thoracic

722.83 Postlaminectomy Syndrome - Lumbar

722.9 Calcification of Intervertebral Cartilage or Disc, Not otherwise specified

722.91 Calcification of Intervertebral Cartilage or Disc of Cervical Region

722.92 Calcification of Intervertebral Cartilage or Disc of Thoracic Region

722.93 Calcification of Intervertebral Cartilage or Disc Of Lumbar Region

723 Cervical Spinal Stenosis

723.1 Cervicalgia

723.3 Cervicobrachial Syndrome

723.4 Brachial Neuritis, Not otherwise specified

723.5 Contracture of Neck, Not otherwise specified

723.6 Panniculitis Affecting Neck

723.7 Ossification Cervical Ligament

723.8 Cervical Syndrome, Not elsewhere classified

723.9 Disorder of Cervical Region, Not elsewhere classified

724 Spinal Stenosis, Not otherwise specified

724.01 Spinal Stenosis f Thoracic Region

724.02 Spinal Stenosis of Lumbar Region

724.09 Spinal Stenosis, Not otherwise specified

724.1 Pain in Thoracic Spine

724.2 LBP [Low Back Pain]

724.3 Cotungo's Disease

724.4 Lumbosacral Neuritis, Not otherwise specified

724.5 Back Pain

724.6 Ankylosis of Lumbosacral Joint

724.7 Disorder of Coccyx, Not otherwise specified

724.79 Coccydynia

724.8 Ossification of Posterior Longitudinal Ligament, Not otherwise specified

724.9 Ankylosis Of Spine, Not otherwise specified

729 Fibrositis, Not otherwise specified

729.1 Fibromyalgia

729.2 Neuralgia, Not otherwise specified

729.4 Fasciitis

729.5 Pain in Limb

Source: Tian TY, Zlateva I, Anderson DR. Using electronic health records data to identify patients with chronic pain in a primary care setting. Journal of the American Medical Informatics Association. 2013; 20: e275-e80.
