# Supplementary material for: The use of complementary and integrative health approaches for chronic musculoskeletal pain in younger US Veterans: An economic evaluation
Source: PLoS One. 2019 Jun 5;14(6):e0217831. doi: 10.1371/journal.pone.0217831 (PMC6550429; doi:10.1371/journal.pone.0217831)
Supplement: S1 Fig — These graphs will not exactly match the results from our model because monthly averages across Veterans, which is what is shown here, are not equivalent to average trends across individual Veterans, which is what our model estimates. However, these graphs are offered to help clarify the healthcare cost components involved. As can be seen, the bulk of healthcare costs are outpatient costs and although some reduction is seen in inpatient costs, the main changes pre- to post-CIH start seem to occur in outpatient costs. Nevertheless, our model was estimated using total costs. (PPTX) [file pone.0217831.s003.pptx]

## Slide 1
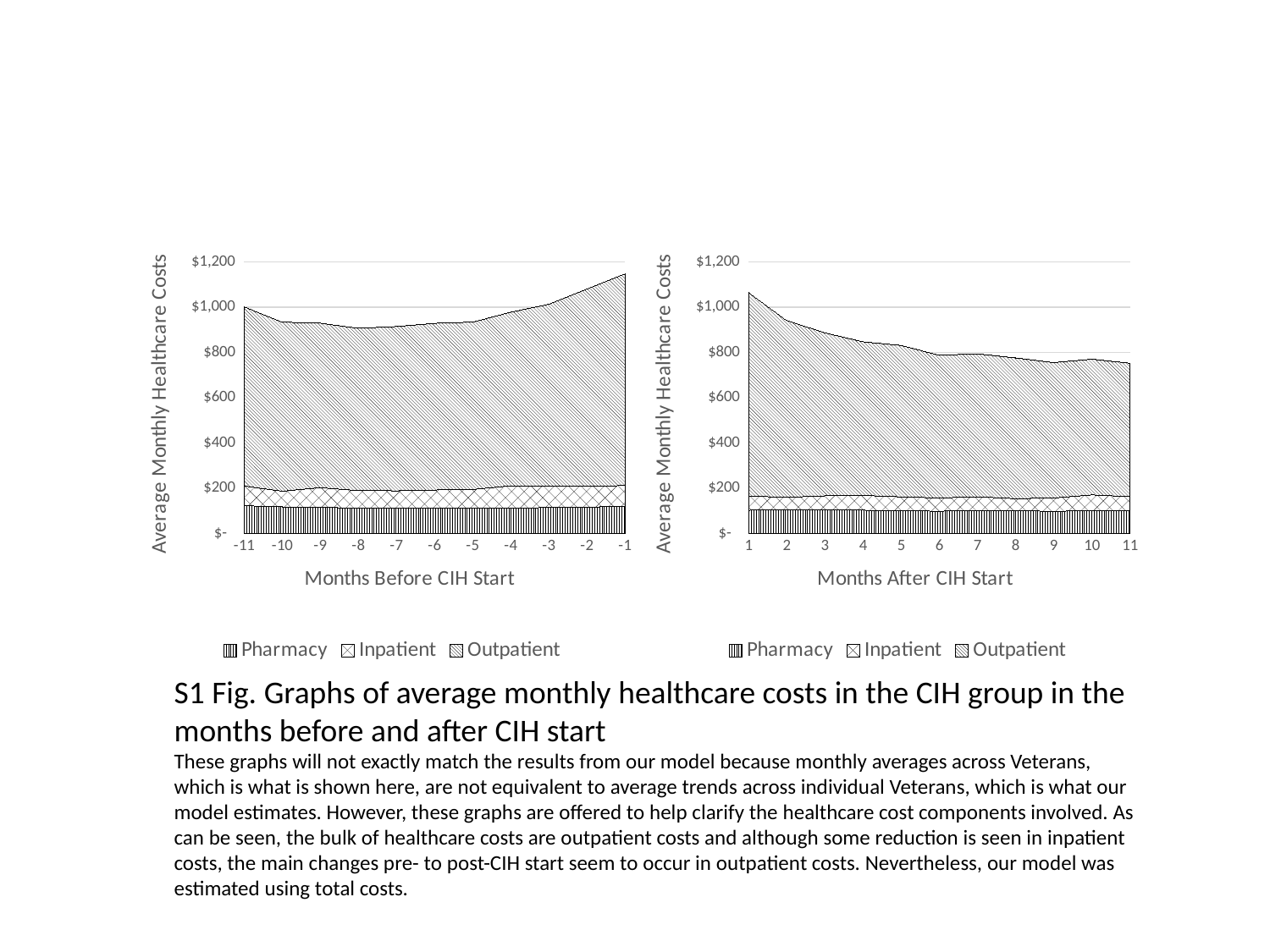

### Chart
| Category | | | |
|---|---|---|---|
| -11 | 122.72375077009201 | 86.97492629289627 | 792.32257604599 |
| -10 | 117.6740974187851 | 69.35925036668777 | 747.3028302192688 |
| -9 | 115.83256721496582 | 86.88551932573318 | 726.7632484436035 |
| -8 | 111.55737191438675 | 77.94561982154846 | 717.6707983016968 |
| -7 | 111.97806149721146 | 76.52116566896439 | 726.2762188911438 |
| -6 | 112.65330016613007 | 79.4791653752327 | 736.8724942207336 |
| -5 | 112.78349906206131 | 81.63032680749893 | 740.0904297828674 |
| -4 | 112.00431734323502 | 98.97341579198837 | 767.453134059906 |
| -3 | 114.41271752119064 | 93.2704508304596 | 805.659830570221 |
| -2 | 116.54003709554672 | 91.49209409952164 | 872.7401494979858 |
| -1 | 119.7604164481163 | 92.14408695697784 | 935.8572363853455 |
### Chart
| Category | | | |
|---|---|---|---|S1 Fig. Graphs of average monthly healthcare costs in the CIH group in the months before and after CIH start
These graphs will not exactly match the results from our model because monthly averages across Veterans, which is what is shown here, are not equivalent to average trends across individual Veterans, which is what our model estimates. However, these graphs are offered to help clarify the healthcare cost components involved. As can be seen, the bulk of healthcare costs are outpatient costs and although some reduction is seen in inpatient costs, the main changes pre- to post-CIH start seem to occur in outpatient costs. Nevertheless, our model was estimated using total costs.
